# Twenty-Eight AM Ismail Oration Healing in Surgery

**DOI:** 10.5704/MOJ.2211.001

**Published:** 2022-11

**Authors:** P Balasubramaniam

**Affiliations:** College of Surgeons, Academy of Medicine of Malaysia, Kuala Lumpur, Malaysia

**The 28th AM Ismail Oration, delivered by Professor P. Balasubramaniam in 2001 has never been published. The Malaysian Orthopaedic Journal is thankful to the College of Surgeons, Academy of Medicine of Malaysia for their permission to publish this lecture in memory of the late Professor P. Balasubramaniam.**

It is an honour to be here today to deliver the AM Ismail Oration and I would like to thank the College of Surgeons and the Academy of Medicine Malaysia for inviting me to deliver the 28th AM Ismail Oration. I feel humbled by the task, for 27 distinguished speakers have preceded me and left their mark. As a past member of your College, I have listened to some of the AM Ismail Orations, and I am acutely aware of the high standards they have set. I shall try to keep up with their tradition.

This oration provides an opportunity for us to pay respect to a great man whom the College is honouring. What can I say of this great man? Tan Sri Abdul Majid Ismail was one hundred percent a Liverpool man steeped in the traditions of Robert Jones, Hugh Owen Thomas, Bryan McFarland, Robert Roaf and many others. Dr AM Ismail was the first Malaysian orthopaedic surgeon. He not only practised but also built-up orthopaedics in Malaysia in the Liverpool tradition. His registrars were trained well by him and sent for further training to Liverpool. After their return, they manned orthopaedic services in various parts of the country. This was a sound system through which Dr AM Ismail ensured quality and standards at a very early stage in the development of orthopaedics in the country. This strong foundation for the development of orthopaedics in Malaysia was possible because of Tan Sri Abdul Majid Ismail’s vision and effort. We therefore fondly call him the father of orthopaedics in Malaysia.

Secondly, Tan Sri Abdul Majid Ismail was an academic man. The term academic is not exclusive to university teachers. Academic ability is in the mind of a man and not in the label. Tan Sri Abdul Majid Ismail was an academic right down to his bones. He, with his wide-ranging intellect and brilliant critical mind could have easily been the first professor of orthopaedic surgery in the country, had he elected to do so. But he elected to be otherwise, for he belonged to an earlier generation of surgeons on whom the demands and challenges were heavy and different. There was no beaten track. He was the trail, and he was also the traveller and therefore as a pioneer, he had to move up and occupy positions from which he could have a wider view and stronger influence to effect changes. Thus, we see him as the founder-president of both the Malaysian Orthopaedic Association and the College of Surgeons of Malaysia. Through these professional bodies, he laid the foundation for the advancement of surgery and orthopaedics in the country. As chairman of the Council of University Malaya, he had a profound influence on tertiary education, research and the founding of other universities in the country.

Later, as Director General of Health Services, he saw to the development of specialist medical services in the country through his creation of medical institutes for various specialities. The various institutes like Institute of Orthopaedics, Institute of Urology and other similar medical institutes bear testimony to his vision and efforts.

Tan Sri Abdul Majid Ismail is thus a multifaceted giant who did not want to stay in the narrow field of orthopaedics longer than was necessary but kept moving forward to develop tertiary education and health services in the country - a duty, I would say, well performed. That was his dharma or duty.

I have chosen the topic, “Healing in Surgery", for my oration. This topic is a broad one which cuts across all the specialities in surgery and would therefore suit all of us this evening. All the previous speakers at this oration have woven three basic strands into their orations - originality of thought, originality of their research work and life-long experience. I shall therefore try to make these the cornerstones of my talk. For a successful outcome of surgery, its science and art of healing have to be taken together. Therefore, my talk is in two parts, first the science of healing and later the art of healing that I practised as a surgeon.

The biology of healing has absorbed my interest during the last thirty years. I shall take you through my experimental studies on healing which were initially done in the University of Malaya and later continued in the National University of Singapore. All the studies taken together would give a glimpse of the problems in healing of tissues and organs.

As a tribute to Tan Sri Abdul Majid Ismail, I would like to mention here that the initial stimulus for my research on collagen and biology of healing was a publication by him in the Journal of Bone and Joint Surgery in 1969^1^. It was on the rupture of patellar ligament after steroid infiltration. He presented his work at a meeting of the Malaysian Orthopaedic Association. As a young lecturer, I was stimulated by that and wanted to find the cause of ligament rupture after steroid infiltration. The belief at that time was that the local action of steroid infiltration was only an anti-inflammatory one. That there could be other effects were not thought of. The late Professor Prathap and I studied the effect of injection of hydrocortisone into rabbit calcaneal tendons^2^. We found that it produced necrosis of collagen in the injected tendon and concluded that similar morphological changes may account for the spontaneous rupture of tendons in patients receiving steroid infiltration. Our study showed that a tendon or ligament injected with steroid into its substance would become weak as a result of necrosis of collagen and therefore is at risk for rupture.

The first study was with light microscopy. To find out the changes at ultrastructural level, electron microscopic(EM) studies^3^ were done on tendons injected with different types of steroids. An invitro study was also done on teased collagen soaked in steroids^4^. The necrosis of collagen after steroid infiltration was confirmed by the EM study and this was found to be at its fibril level. The breakdown of collagen happened fast within a few seconds of injection of the steroid. Similar changes were also observed in the invitro study, pointing to a chemical effect as a possible cause of necrosis of collagen. The word collagen means “to form glue". Collagen can be denatured by physical, chemical or biological means. Heat above 60°C, potassium thiocyanate or guanidine hydrochloride will denature collagen. It is likely that local infiltration of tendon or ligament with steroid causes denaturation of its collagen to form glue by a similar chemical action. They all break the intermolecular and intramolecular bonds of the triple helix of collagen to produce dimers, monomers and finally gelatine.

Following the collagen study, I moved on to a comparative study of healing in three different types of tendon lengthening. We found that the healing in the three different types of tendon lengthening were similar. In contrast to other studies, no intrinsic tenoblastic activity was seen and the repair tissue was found to come from the surrounding tissues^5^. This type of healing was also similar to the repair of necrotic lesion in tendon produced by injection of steroid in my previous study^2^.

## Phosphate Supplements and Healing of Fractures

Phosphate ion takes part in mineralisation of bone. High phosphate supplements are therefore believed to be useful in fracture healing and osteoporosis. An experimental study was done while I was on sabbatical leave in the Mayo Clinic. It was on the healing of fracture tibia of rabbits fed on high phosphate supplements^6^. Our radioisotope studies showed that large amounts of calcium were retained in the body of rabbits fed on high phosphate supplements. The high retention of calcium was thought to be in the bone through new bone formation. But estimation of calcium in different soft tissues of rabbits fed on phosphate supplements showed it to be otherwise. The calcium was found to be not in the bones but in soft tissues like liver, kidney and aorta which showed pathological calcification. The fractured tibia of animals fed on high phosphate supplements showed no increase in bone formation but a porotic callus and porosis of normal bone. These findings were quite different from what we set out to show. The use of phosphate supplements in fracture healing or metabolic bone disease must not therefore be taken lightly for they might induce pathological calcification in soft tissues and increase the porosis of bone.

## Healing of Non-Vascularised Diaphyseal Bone Transplants

My interest in this study arose from claims made in the literature that a bone gap more than two inches needed microvascular bone transfer to bridge it. But, before microvascular transfer came into common use, we used non-vascularised fibula bone graft to bridge large gaps after excision of giant cell tumour of radius or the Papineau method to fill large gaps after a debridement for chronic osteomyelitis. These two methods worked well, and the grafted bone was quite strong by about eight to nine months. An experimental model was designed where two thirds (4 cm) of a cat’s tibia was excised and replanted in four different ways in groups of the animals. In the first group, the periosteum of the segment was left intact while in the second group, the diaphyseal segment had the periosteum removed. In group three, the segment had no periosteum, and its medullary canal was also plugged with a silastic rod to cut off blood supply. The fourth group was similar to group three but in addition the diaphyseal segment was isolated from the surrounding muscles by wrapping the segment of bone with a silastic sheet. This work was for a MD thesis^7^, and I was the supervisor. We found that the fracture of the diaphyseal segment healed in the first three groups by callus encasement. In the fourth group where the bone segment was isolated from surrounding muscles, there was little or no healing of the fracture. This experiment shows that even a large diaphyseal segment of bone completely denuded of its blood supply and periosteum heals first by callus encasement and gets incorporated later by creeping substitution provided there are enough muscles around it.

## Fracture Healing in the Foetus

The study of fracture healing in the foetus came about after a failure of my study in foetal rabbits to produce congenital constriction rings^8^. The study was therefore switched to a comparative study on the healing of closed and open fractures in 15-day old foetus of rabbits which have a period of gestation of 30 days. It was done while I was in the University of Malaya.

After hysterotomy of a pregnant rabbit, its amniotic membrane was opened and a leg of the foetus was delivered outside the uterus. It was fractured and the fractured limb was returned to the uterus and the hysterotomy was then closed. Pregnancy was allowed to continue till term and histological studies of the fractured legs were done. Closed fractures were found to heal by a process very similar to that of endochondral bone formation in a foetus^9^. Inflammatory response during the fracture healing in a foetus was found to be minimal. Similar absence of an inflammatory response during healing of foetal skin was also reported by Somasundaram and Prathap earlier. We must note that Professor Somasundram’s AM Ismail Oration was on “Foetal Surgery - Experimental Model To Therapeutic Reality”^10^.

In my study, open fractures of tibia in the foetus were found not to heal at all. There was no inflammatory response either. Cells of cambial layer of periosteum of the broken ends of bone in an open fracture were seen to multiply in large numbers without producing any callus. There was no attempt at bridging the skin or the bones in an open fracture of a rabbit foetus. The failure of healing of an open fracture in the foetus could be due to the fracture haematoma being washed off by amniotic fluid, giving no chance for the cascade of events in healing set in motion by mediators in a fracture haematoma. The failure may also be due to other causes and this needs to be studied further.

## Nerve Healing - Bridging of Large Gaps in Nerves

Managing nerve injury with gaps that cannot be bridged by direct suture is a difficult problem. Nerve grafts though useful have their limitations in terms of availability, size and fibre diameter. Sural nerve, the common donor material, does not meet the requirements of bridging large nerves like median or ulnar nerves. Cable grafts on the other hand produce excessive fibrosis, scarring and poor axonal regeneration. Therefore, an alternative bioprosthesis becomes an attractive choice.

Basal lamina tubes from nerves as well as skeletal muscles have been experimentally shown to guide regenerating axons into the distal segment of a cut nerve. Even non-biological conduits like silicone tubes or collagen membrane have also been shown to produce similar results. These conduits serve as temporary bridges till the Schwann cell tubes from regenerating nerve fibres take over.

Muscle basal lamina tubes have been used in humans to aid sensory recovery in digital nerves^10^. An experiment was therefore devised to find out whether muscle basal lamina tubes would also aid motor recovery. Two cm of the sciatic nerve of rabbits were excised and the gap was bridged by coaxial suture of basal lamina tubes prepared from hamstring muscle. The basal lamina tube graft was prepared by freezing the excised muscle in liquid nitrogen and thawing it later in distilled water.

Before sacrifice, nerve conduction studies were done and fluorescent dyes like fast blue and fluorogold were injected into the nerve distal to the graft to trace their transport along regenerated axons to cell bodies in dorsal root ganglion and anterior horn cells of spinal cord. EM studies showed regenerating myelinated axons within the muscle basal lamina tube grafts within a week. By 12 weeks, 80% of dorsal root ganglia and 30% of anterior horn cells showed fluorescence of the injected dye thus indicating that axonal regeneration and continuity with the nerve distal to the graft had been established through the basal lamina tube grafts.

Motor conduction velocity of grafted nerve was 53% of the normal by 12 weeks. These results show that muscle basal lamina tube grafts help in motor as well as sensory recovery^11^. Basal lamina tube graft from a muscle is therefore a surgical option to bridge large gaps in nerves that are not suitable for direct repair.

## Spinal Cord Regeneration

My attempt at experimental regeneration of spinal cord with foetal neural tissue transplants from the brain of a 10-day rabbit foetus was a failure, even after one year of trial. Managing bladder and anaesthetic ulcers of a paraplegic rabbit was a problem and all the rabbits died within 4 to 6 weeks of the experiment. Even an experimental model with hemisection of cord did not work for these animals too died. I realised now that larger animals and microtechniques would have been better. The old concept that a neurone is a permanent cell is now changing. When stem cell transfer and tissue engineering techniques for spinal cord are perfected, spinal cord regeneration could become a reality.

## The Future of Healing by Surgery

I have so far looked at some aspects of healing through my work on musculoskeletal tissues. They, taken together, only provide fragments of knowledge and partial answers for a vast problem, the biology of healing which is as old as and is related to evolution of species. It is a pre-programmed event where the healing process is a cascade of cell division, migration and differentiation driven by locally released mediators or growth factors. Each tissue theoretically has a full potential for healing and repair, but this has through specialisation, become modified or lost in certain tissues like articular cartilage, brain, spinal cord and muscles. Their genetic switches for healing can be turned on provided we find out how to do them.

There appears to be a fundamental difference between the healing and repair of tissues in a foetus and an adult. The inflammatory response seen during repair is very much an adult phenomenon. It does not seem to occur during the repair of skin or bone in a foetus. The inflammatory response during healing, though it prevents infection produces fibrosis which can impair the quality of healing. Can we borrow ideas from healing in the foetus and use foetal cells and growth factors to resurface or repair articular cartilage?

Healing and repair of osteoarthritis, stroke, head injury, or paraplegia will soon be a reality through the use of stem cells, tissue engineering and growth factors. The problem is in interphasing them at the molecular, biological and physical level. Once this can be overcome, it will revolutionise our current methods of healing by surgery and the scalpel will become less and less in use by the surgeon.

Healing by surgery will continue to change with advances in scientific knowledge and technology. The current minimally invasive surgical techniques will be overtaken or improved by molecular biology techniques in the future. We, surgeons, have to keep abreast with the changes if we are to survive.

## The Art of Healing in Surgery

Now, I would like to move on to the second part of my oration — the art of healing in surgery. I would relate it the way I practised it. What I say may not be different from what you and I do. It will only reaffirm what we surgeons do to heal. The science of healing alone is not enough. It has to be woven into the art of healing for a successful outcome. How can I paint the art of healing in surgery on a broad canvas of this oration? It has to be made of different strokes to get a composite picture. The first few strokes are on the necessary attributes of a surgeon - competence, commitment, compassion, communication and case records. Of the five Cs that I have listed, competence comes first, for the rest without competence, will be of little or no value. Similarly effective communication and good case records are part of surgical care, though we usually tend to neglect them. They are becoming more and more important with increasing number of medico-legal litigations.

Secondly, I feel that an effective planning and conduct of pre-operative, perioperative and post-operative care is vital. The success of healing after surgery depends on all these three levels of care, with good judgement and lot of attention to details.

In planning surgery, it is important to bear in mind that no surgery is minor. Every surgery is major and each surgery for me was an Olympic race. For every case, I would start thinking about it and rehearse the mental images of various steps of surgery a day or two before it. I would also image in my mind the positioning, draping, incision, exposure and handling of possible difficulties. This was also supplemented by references to anatomy and operative surgery textbooks. Surgery preceded by this manner of preparation was very anatomical and precise. Patients were seen by me personally the previous day, case notes made, site marked, and the details of operation discussed with a sense of reassurance while holding their hands. The next morning, I would greet the patient in the induction room. Preparation in this manner made even the most difficult surgery, easy. But in spite of all these preparations, things did go wrong sometimes. I have made mistakes and learnt from them. The opposite is also true. Sometimes, in spite of everything going wrong in the operating theatre, the patient is alive, well and thankful the next day when you see him in the ward. You wonder how this is possible, except by divine grace and intervention.

There is a mind-body and spirit in healing, and they have to be dealt together. We may heal a disease by surgery but not the illness of the patient. Disease is not equal to illness which is a patient’s experience of disease. We surgeons are often blind to how patients react emotionally to their disease. Inattention to the emotional reality of illness neglects a growing body of evidence showing that patients’ emotional status play a significant role in their recovery. Modern surgical care with high technology often lacks emotional intelligence. Emotional intervention should continue to be a part of our surgical care even with scientific advances.

I would sum up by saying that no surgery for me was minor. Every surgery was an Olympic race which needed my careful planning and execution with lot of attention to details and taking into account the body-mind and soul of the patient. Surgery is a science which becomes an art through its practice. For a satisfactory outcome in healing after surgery, the science has to be balanced with the art coming not only through the surgeon’s hands but also through his head and heart.
